# Peliosis Hepatis With Intrahepatic Hemorrhage: Successful Embolization of the Hepatic Artery

**DOI:** 10.1155/2000/94813

**Published:** 2000-01

**Authors:** K. O'Riordan, A. Blei, R. Vogelzang, A. Nemcek, M. Abecassis

**Affiliations:** 1 Department of Medicine Northwestern University Medical School Chicago Illinois USA; 2 Department of Radiology Northwestern University Medical School Chicago Illinois USA; 3 Department of Transplantation Northwestern University Medical School Chicago Illinois USA; 4 Northwestern University Medical School 303 E. Superior Street Suite 528 Chicago Illinois 60611 USA

## Abstract

Peliosis hepatis is defined as the appearance of blood filled lakes in the hepatic parenchyma. It has been associated with various pharmacological agents and infections. Treatment has been primarily symptomatic and includes discontinuation of offending medications, partial hepatectomy or occasionally liver transplantation. We report a 58 year old white female on hormone replacement therapy who developed symptomatic peliosis hepatis and underwent successful superselective hepatic artery embolization with control of bleeding.


*HPB Surgery*, 2000, Vol. 11, pp. 353–358
Reprints available directly from the publisher
Photocopying permitted by license only

© 2000 OPA (Overseas Publishers Association) N.V.
Published by license under
the Harwood Academic Publishers imprint,
part of The Gordon and Breach Publishing Group.
Printed in Malaysia.

### *Case Report*

### Peliosis Hepatis with Intrahepatic Hemorrhage: Successful Embolization of the Hepatic Artery

K. O'RIORDAN[a], A. BLEI[a], R. VOGELZANG[b], A. NEMCEK[b] and M. ABECASSIS[c,*]

[a] *Departments of Medicine, Northwestern University Medical School, Chicago, Illinois;*
[b] *Department of Radiology, Northwestern University Medical School, Chicago, Illinois;*
[c] *Department of Transplantation, Northwestern University Medical School, Chicago, Illinois*

*(Received 22 December 1996; In final form 5 July 1998)*

**Peliosis hepatis is defined as the appearance of blood filled lakes in the hepatic parenchyma. It has been associated with various pharmacological agents and infections. Treatment has been primarily symptomatic and includes discontinuation of offending medications, partial hepatectomy or occasionally liver transplantation. We report a 58 year old white female on hormone replacement therapy who developed symptomatic peliosis hepatis and underwent successful superselective hepatic artery embolization with control of bleeding.**

*Keywords:* Peliosis hepatis, intrahepatic hemorrhage, hepatic embolization

### INTRODUCTION

Peliosis hepatis was initially described in 1916 by Schoenlank [1] and is defined as the extravasation of blood into the hepatic parenchyma. The first English literature description was in 1950 by Zak [2]. It is characterized by the presence of multiple, randomly distributed, blood filled cystic areas of variable size. It causes St George's disease in cattle [3], a wasting disease with similar hepatic lesions to man. Most cases are diagnosed post mortem. There is no hepatic zonal predominance and peliosis may also affect the spleen, lymph nodes, bone marrow, lungs, kidneys, adrenals, stomach and small bowel [4].

Fatal hepatic rupture may be consequent upon free sinusoidal communication resulting in rupture of other organs including the spleen. In all cases of fatal hemorrhage patients were concomitantly taking exogenous steroids [5]. What is unknown is the rate of progression of peliosis hepatis with time. Successful treatment with arterial embolization of a ruptured hepatic peliotic cavity has not been previously reported.

*Author for correspondence: Michael Abecassis, MD, Northwestern University Medical School, 303 E. Superior Street, Suite 528, Chicago, Illinois 60611. Tel.: (312) 908-8900, Fax: (312) 908-9194.

### CASE HISTORY

A 58 year old white female was evaluated for abdominal pain and an expansile hepatic mass. She underwent a hysterectomy and bilateral oophorectomy seven years before presentation after which she was commenced on conjugated estrogen 0.625 mg/day. Other medications included aspirin 325 mg/day for arthritis and amlodipine 5 mg/day for hypertension. In September 1994, she developed a sudden, severe, sharp pain over the right shoulder joint with radiation to the anterior chest wall and right arm associated with dizziness and culminating in a presyncopal episode. Initial evaluation found her to be bradycardic and hypotensive. Examination revealed right upper quadrant tenderness. Laboratory evaluation showed a hemoglobin of 1.22 m mol/L with normal electrolytes, glucose and liver chemistries. An urgent CT scan of the abdomen and pelvis (Fig. 1) revealed a large blood-filled area abutting upon the right lobe of the liver associated with capsular distension and with adjacent necrosis secondary to parenchymal compression. Hepatic angiography (Fig. 2) revealed the presence of several, small, peliotic cavities with preferential right lobe involvement. She was resuscitated with two units of packed red blood cells. Over the next 48 hours she developed worsening abdominal pain and an enlarging hematoma on repeat CT scanning. She required transfusion of packed red cells.

Physical examination revealed tender hepatomegaly with the lower border palpable in the right iliac fossa. There was no splenomegaly. The stool was green, hemoccult negative.

Laboratory evaluation revealed a hemoglobin of 1.77 m mol/L, a normal white cell and platelet count. Prothrombin time, electrolytes, BUN and creatinine were normal. Repeat liver chemistries revealed an AST 139 U/L, ALT 151 U/L, bilirubin 46.7 μmol/L, ALP 75 U/L, GTP 100 U/L with an LDH of 262 U/L and an albumin of 35 gm/L. Urinalysis showed 3 to 5 white cells. Further laboratory evaluation revealed a fibrinogen of 21.1 μmol/L, fibrin degradation products of < 20 mg/L. Individual clotting factors were normal and a bleeding time was 6 minutes. Thyroid function tests were normal and she was HIV negative.

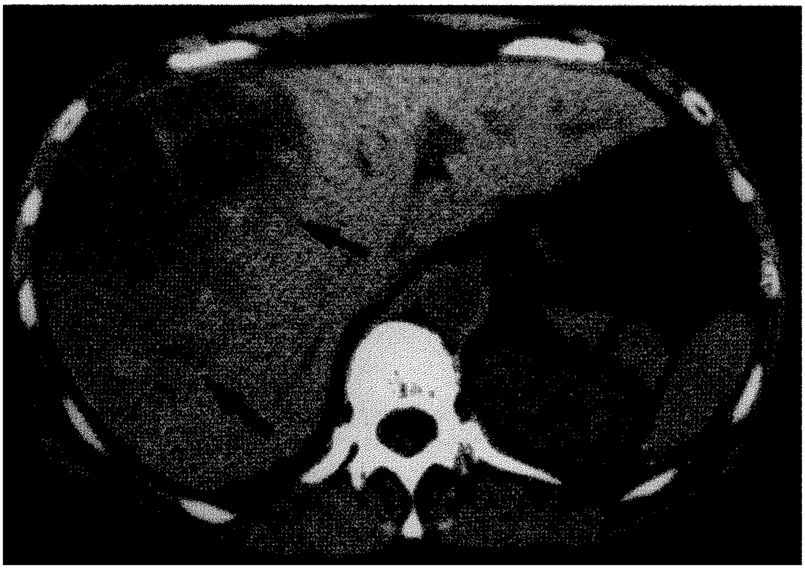

FIGURE 1 Noncontrast CT scan showing large hepatic hematoma with heterogenous density (arrows).

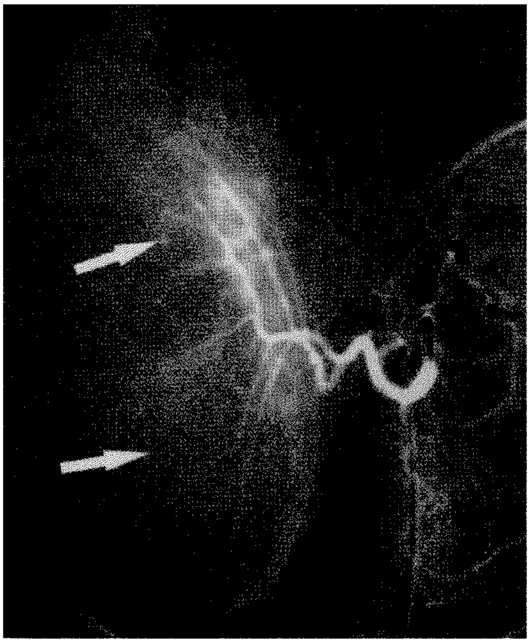

FIGURE 2 Hepatic arteriogram demonstrating mass effect of large hepatic hematoma upon the right lobe as well as multiple small areas of contrast accumulation consistent with peliotic cavities (arrows).

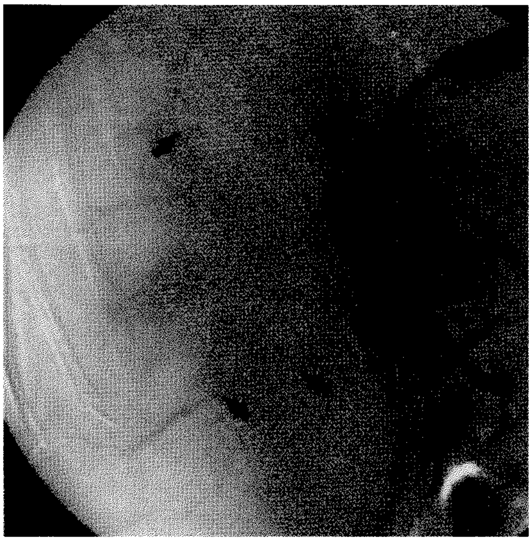

FIGURE 3 Selective hepatic arteriogram after superselective embolization of the right hepatic artery with multiple intrahepatic vascular occlusions (arrows).

The patient underwent repeat CT scanning which revealed an enlarging hepatic hematoma. Repeat hepatic angiography revealed the presence of bilateral small peliotic cavities with preferential right lobe involvement. The patient was treated with prophylactic antibiotics and underwent successful superselective embolization of the right hepatic artery. Following celiac arteriography, the common hepatic artery was subselectively catheterized with a 5 French catheter for hepatic arteriography. After performance of the hepatic arteriogram, the right posterior segmental artery was coaxially superselectively catheterized with a Tracker-18 microcatheter (Target Therapeutics, Fremont, CA) and embolized with 250 to 500 μm diameter polyvinyl alcohol foam particles (Cook, Inc., Bloomington, IN). Follow-up arteriography showed complete occlusion of the posterior division with preservation of flow in the anterior segmental branch (Fig. 3). Repeat CT scans in the hospital showed a slight improvement in hematoma size and sequential scans over the following 9 months showed greater than 80% resolution of the hematoma (Fig. 4) with scarring and fibrofatty replacement and with normalization of the adjacent hepatic parenchyma and the patient has remained symptom free and continues off all medications.

### DISCUSSION

Peliosis hepatis has been associated with chronic wasting diseases such as tuberculosis or carcinoma, prolonged use of birth control pills and anabolic steroids, AIDS, chronic vitamin A administration, pemphigus and septic shock [5–7]. The pathogenesis is multifactorial and includes hepatocyte necrosis adjacent to the peliotic cavities with resultant destruction of the reticulin framework allowing cysts to form from adjacent sinusoidal inflow of blood. Furthermore, there may be blockage of liver blood outflow at the junction of the sinusoids and the centrilobular veins [8,9]. Direct lesions of the sinusoidal barrier have also been found [7].

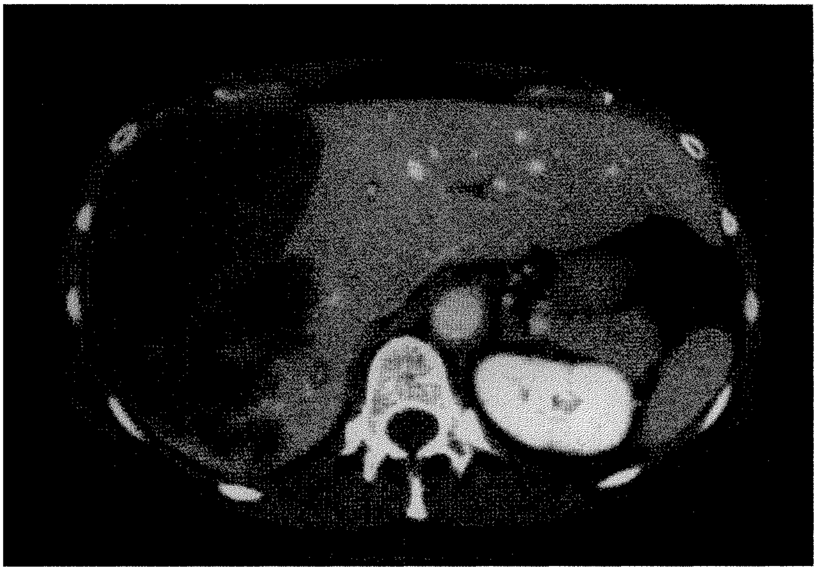

FIGURE 4 Follow-up CT scan 10 days after embolization showing low density hematoma and irregular low density regions in the posterior aspect of the right lobe secondary to hepatic infarction and necrosis.

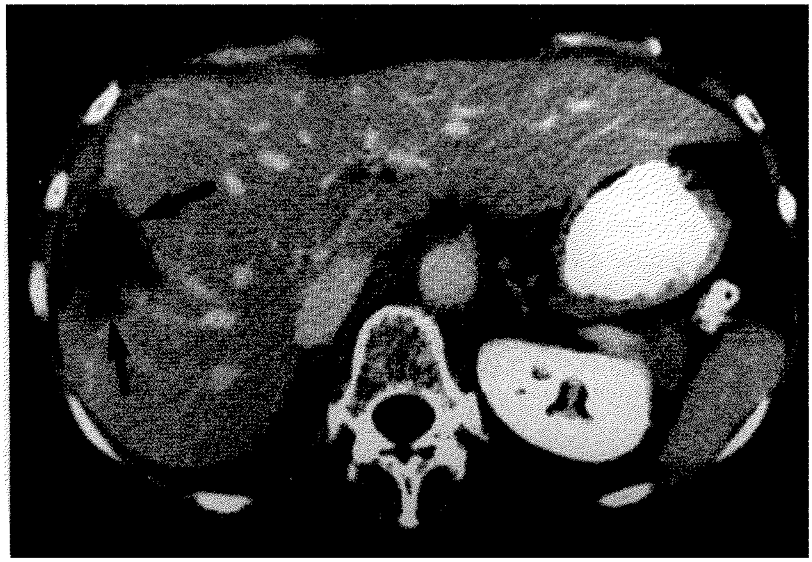

FIGURE 5 Follow-up CT scan one year after embolization with almost complete resolution of hematoma and small low density residual area (arrows).

Recently, a bacillary peliosis hepatitis has been reported in association with AIDS caused by Rochalimaea henselae [10].

Clinically when small and few premonitory symptoms are present, few signs occur as the injury is primarily sinusoidal and not parenchymal. Commonly, patients present with hepatomegaly, hepatic dysfunction or signs of hepatic rupture if the lesions are large and numerous. Death may result from intraperitoneal hemorrhage or hepatic failure. Rarely, peliosis hepatis may present with cirrhosis, portal hypertension, cholestasis or the hepatorenal syndrome.

The differential diagnosis of these lesions includes cavernous hemangioma, focal nodular hyperplasia and hepatic adenoma, although definitive diagnosis ultimately rests on the characteristic liver tissue. In peliosis hepatis, ultrasound examination may show areas of mixed hyper and hypoechoic lesions of the liver and CT scanning frequently reveals patchy low density areas [11]. Characteristic angiographic features are visualization of peliotic nodules of various sizes in the late arterial phase which become more prominent in the parenchymal and venous phases [11, 12]. Similar findings may occasionally be seen in hemangiomas but these usually have a more irregular configuration. Wedged hepatic arteriography reveals opacification of the peliotic cavities sometimes clustered around the hepatic vein radicals which opacify prior to the hepatic sinusoids implying a direct communication with the hepatic venules. Our patient's radiologic features are typical of those described in peliosis hepatis.

Histology is the only definitive way to make the diagnosis but was considered hazardous in our patient. The peliotic cavities are randomly distributed throughout the liver without any zonal preference. Most are sharply outlined cystic spaces filled with blood, but lined by endothelial or Kupffer cells. Two stages have been described by Yanoff and Rawson [13]. The phlebectatic variant is characterized by the presence of vascular spaces with regular contours of uniform sizes in a predominately centrilobular location which communicate with the central veins and compress the adjacent parenchyma. The parenchymal variant demonstrates irregular, diffuse, non-uniform blood filled spaces with no endothelial lining or communication with the central veins or compression of the adjacent parenchyma, but there is evidence of necrosis. The above classification, however, is controversial and it is frequently difficult to distinguish between the two types and both lesions may occur simultaneously.

Traditional treatment involves discontinuing offending medications, operative intervention or ultimately liver transplantation. Spontaneous resolution is unusual [14, 15]. Angiography with embolization has long been used as both a diagnostic and therapeutic modality in various gastrointestinal diseases including the diagnosis and therapy of patients with gastrointestinal bleeding. Hepatic arterial embolization has similarly been used both as a therapeutic modality as well as for the delivery of chemotherapeutic medications in the treatment of hepatocellular carcinoma [16]. Hayward *et al.*, described the successful treatment of a patient with two episodes of bleeding secondary to peliosis hepatis with hepatic dearterialization [17].

### SUMMARY

Our patient presented with a hepatic hematoma secondary to rupture of a peliotic cavity. A percutaneous or transjugular liver biopsy was not performed because of concern of further clinical deterioration. Fortunately, the hematoma was partially controlled by the liver capsule allowing successful treatment with superselective hepatic arterial embolization of a ruptured hepatic peliotic cavity. The exact etiology of the peliotic cavities in our patient is unclear but is most likely secondary to the use of estrogens. With one year of follow-up, the patient has had no recurrence of symptoms and an 80% reduction in hepatic hematoma size. The ultimate fate of the remaining peliotic cavities in the left lobe of the liver is unclear and the value of prophylactic radiologic screening of these peliotic cavities is unknown.

### CONCLUSION

In summary, peliosis hepatis has been associated with the use of synthetic estrogens. It frequently has a fatal outcome secondary to rupture and intraperitoneal hemorrhage. Hepatic arterial embolization offers an alternative to operative intervention in patients with contained peliotic cavities.

### References

[1] Schoenlank, F. W. (1916). Ein Fall von Peliosis hepatis. *Virchows Arch. Pathol. Anat.*, **222**, 358–364.
[2] Zak, F. G. (1950). Peliosis hepatis. *Am. J. Pathol.*, **26**, 1–15.
[3] Seawright, A. A. and Francis, J. (1971). A specific liver lesion in St. George disease of cattle. *Aust. Vet. J.*, **47**, 91–99.
[4] Ichijima, K., Kobashi, Y., Yamabi, H. *et al.* (1980). Peliosis hepatis: an unusual case involving multiple organs. *Acta Pathol. Jpn.*, **30**, 109–120.
[5] Naeim, F., Cooper, P. H. and Semion, A. A. (1973). Peliosis hepatis: possible role of anabolic steroids. *Arch. Path.*, **95**, 284–285.
[6] Bagheri, S. A. and Boyer, J. L. (1974). Peliosis hepatis associated with androgenic anabolic steroid therapy: a severe form of hepatic injury. *Ann. Int. Med.*, **81**, 610–618.
[7] Zafrani, E. S., Cazier, H., Baudelot, A. M. and Feldmann, G. (1984). Ultrastructural lesions of the liver in human peliosis: a report of 12 cases. *Am. J. Path.*, **114**, 345–359.
[8] Zafrani, E. S. and Feldmann, G. (1988). Primary lesions of the hepatic sinusoid. In: Bioulac-Sage, P. and Balabaud, C. Eds. Sinusoids in human liver: health and disease. Rijswijk, The Netherlands: The Kupffer Cell Foundation, pp. 207–222.
[9] Degott, C., Rueff, B., Kreis, H., Duboust, A., Potet, F. and Benhamou, J. P. (1978). Peliosis hepatis in recipients of renal transplants. *Gut*, **19**, 748–753.
[10] Perkocha, L. A., Geaghan, S. M., Yen, T. S. B. *et al.* (1990). Clinical and pathological features of bacillary peliosis hepatis in association with human immunodeficiency virus infection. *NEJM*, **323**, 1581–1586.
[11] Tsukamoto, Y., Nakata, H., Kimoto, T. *et al.* (1984). CT and angiography of peliosis hepatis. *AJR*, **142**, 539–540.
[12] Pliskin, M. (1975). Peliosis hepatis. *Radiology*, **114**, 29–30.
[13] Yanoff, M. and Rawson, A. J. (1964). Peliosis hepatis: an anatomic study with demonstration of two varieties. *Arch. Path.*, **77**, 159–165.
[14] Simon, D. M., Krause, R. and Galambos, J. T. (1988). Peliosis hepatis in a patient with marasmus. *Gastroenterology*, **95**, 805–809.
[15] Groos, G., Arnold, O. H. and Brittinger, G. (1974). Peliosis hepatis after long-term administration of oxymetholone. *Lancet*, **1**, 874.
[16] Ensminger, W. D. and Gyves, J. W. (1983). Clinical pharmacology of hepatic arterial chemotherapy. *Semin Oncol.*, **10**, 176–182.
[17] Hayward, S. R., Lucas, C. E. and Ledgerwood, A. M. (1991). Recurrent spontaneous intrahepatic hemorrhage from peliosis hepatis. *Arch. Surg.*, **126**, 782–783.

### PELIOSIS HEPATIS: A WORD OF CAUTION

Peliosis hepatis as described in this case report is mostly observed in men on androgenic–anabolic steroid therapy, in women taking oral contraceptives and in patients in an infectious setting. Its diagnosis is based on hepatic biopsy material even if a radiological presentation could be suggestive of such a possibility. Hemorrhagic complications are rarely observed in peliosis hepatis but may have a fatal outcome. Among potential therapies, embolization now constitutes a first step before surgery and ultimately liver transplantation. However, until now, in such a setting, prophylactic embolization can not be recommended. Such a treatment must indeed be restriced to cases showing active hemorrhage. Since there is always a risk that the embolization procedure can not be superselective and may thus induce extensive liver necrosis and its potential complications.

Dr. Y. Horsmans
Hepato–Gastoenterology Unit
St Luc University Hospital
10, Av. Hippocrate
Bruxelles120
Belgium.

